# Histopathological changes in the human larynx following expanded polytetrafluroethylene (Gore-Tex^®^) implantation

**DOI:** 10.1186/1472-6815-5-1

**Published:** 2005-01-21

**Authors:** K Rajkumar, HS Khalil, M Elloy, E Sheffield, DL Baldwin

**Affiliations:** 1 Departments of Otolaryngology/Head and Neck Surgery, Derriford Hospital, Plymouth, UK; 2 Departments of Otolaryngology/Head and Neck Surgery, Southmead Hospital, Bristol, UK; 3 Department of Pathology, Frenchay Hospital, Bristol, UK

## Abstract

**Background:**

Expanded polytetrafluroethelyne (e PTFE, Gore-Tex^®^) has been advocated as an implant material for medialization of the vocal fold. Animal studies involving rabbits and a porcine model have demonstrated host tolerance of the implant. There have been no reports describing the histological changes in a human laryngectomy specimen with a Gore-Tex implant.

**Case presentation:**

The histological findings in a laryngectomy specimen of a patient previously implanted with e PTFE for medialization of a paralyzed vocal fold following excision of a vagal neurofibroma were studied.

Histopathology revealed a mild foreign-body giant cell granulomatous reaction with some associated fibrosis. The granulomatous response was limited to the periphery of the Gore-Tex and although it closely followed the profile of the material it did not encroach into or significantly break up the material. There was no significant neutrophilic or lymphocytic inflammation.

**Conclusions:**

Our findings are consistent with the animal models confirming that Gore-Tex implantation does not result in a significant granulomatous reaction in the human larynx over a 13-month period. Moreover, there is no evidence of resorption or infection. Further, the lack of lymphocytes in association with the granulomas indicates that there is no significant immunological hypersensitivity. Histologically, the slight permeation by connective tissue is similar to that seen in Gore-Tex vascular and cardiac implants. The degree of the slight giant cell response appears to be dependent on the profile of the material; a sharp edge incited more of a response than a flat surface.

## Background

Unilateral vocal fold paralysis is symptomatic when it results in failure of the mobile vocal fold to approximate the paralyzed vocal fold during adduction. Despite the lack of movement, the paralyzed vocal fold will often contact the contra lateral mobile vocal fold permitting adequate glottic closure [1].

Medialization laryngoplasty is a common procedure used to restore glottic competence. This procedure was popularized by Ishiki and initially used in patients with vocal fold paralysis. In recent years, the indications for this procedure have expanded to include most forms of glottic incompetence, including the use of bilateral medialization in mobile vocal folds for vocal fold bowing and atrophy [2].

Although medialization is now widely accepted, the choice of implant material is still a subject of controversy. The ideal vocal fold injection material would be readily available, have excellent biointegration with no or minimal immunologic response, has an excellent biomechanical in vivo match to the injection site tissues, and is deliverable through a fine-gauge needle. Such a material does not presently exist.

Teflon (Polytetrafluoroethylene) was the first modern material used for vocal fold injection. Long-term results, unfortunately, have shown an unacceptably high rate of granuloma formation associated with Teflon injection. This results in a gradual degradation of the injected material by foreign body giant cells and fibroblastic proliferation in the location of the implant [3]. Autologous tissues such as fat or fascia are well described. These tissues have the benefit of medializing the vocal fold with minimal tissue reaction [4,5]. Collagen injection has been used with some success, its major drawback is the need for serial injections in some cases [2].

Recently, an expanded polytetrafluroethelyne implant (e PTFE, Gore-Tex^®^) has been advocated as an implant material for medialization of the vocal fold [6]. This material has been widely used in cardiac- and vascular surgery and soft tissue augmentation with minimal complications [2]. Cashman et al published a histological study of changes in the rabbit larynx in response to Gore-Tex implantation. They found evidence of a foreign body giant cell reaction and a thin rim of fibrous tissue surrounding the implant. Although the fibrous capsule invaginated between the folds of the implant, it did not appear to grow into the implant itself. The implant was secure in the soft tissue with no migration or spontaneous extrusion noted [2].

## Case presentation

A 64-year-old female patient underwent transcervical excision of a right vagal neurofibroma. This was complicated by right vocal fold paralysis with dysphonia and aspiration. Six months later, the patient underwent fat injection for medialization of the right vocal fold. After an initial improvement, the patient suffered a recurrence of the aspiration, chest infections and dysphonia.

**Figure 1:**
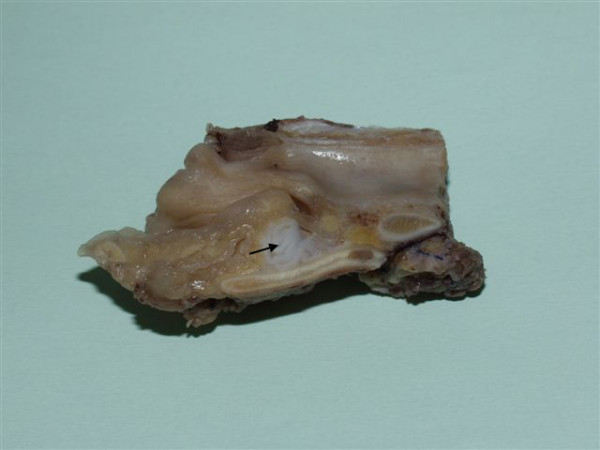
Laryngectomy specimen showing Gore-Tex (arrow) as a pale nodule in the paraglottic space lying beneath an intact mucosa.

The patient subsequently underwent right medialization using Gore-Tex implant (Gore Thyroplasty Device, Medtronics, Xomed^®^) with cricothyroid approximation. Videofluroscopy and Speech assessment confirmed an improvement in aspiration and speech. Six months later, her symptoms recurred with subsequent aspiration pneumonia. A decision was made after lengthy counseling to proceed with a laryngectomy 13 months following the implantation of Gore-Tex. Figure 1 shows the Gore-Tex in the laryngectomy specimen.

### Histopathology

**Figure 2:**
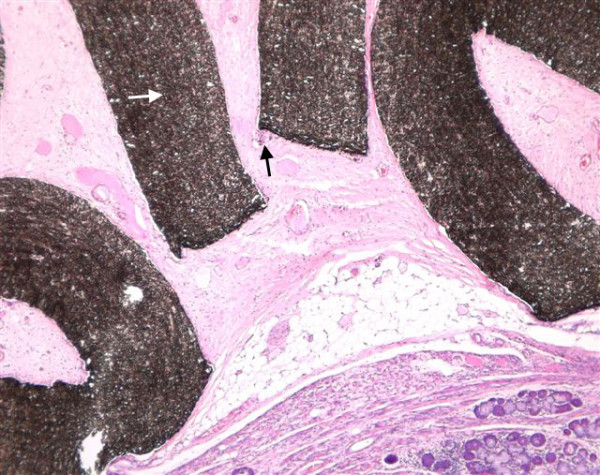
Low power magnification showing broad interlacing bands of Gortex material (white arrow) surrounded by mild foreign-body giant cell granulomatous reaction with some associated fibrosis (black arrow).

**Figure 3:**
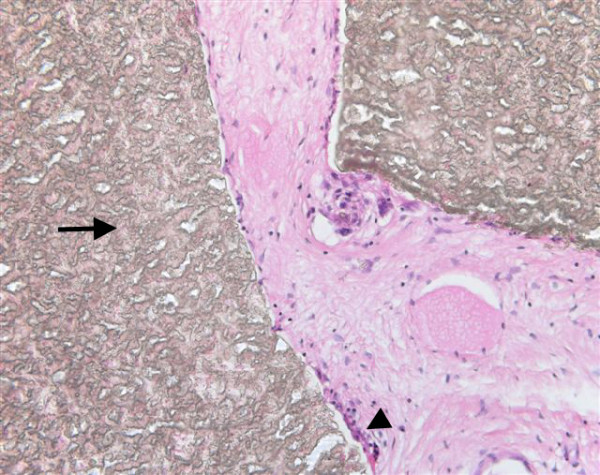
High power magnification showing only a small number of multinucleated foreign-body type macrophage giant cells (arrowhead) associated with the periphery of the Gortex bands (arrow). There is little evidence of breakdown of Gore-Tex.

**Figure 4:**
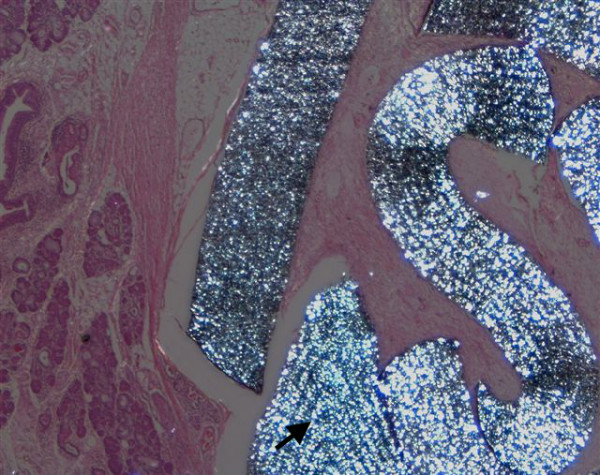
Gore-Tex under crossed polaroids showing birefringence (arrow).

Light microscopy revealed a mild foreign-body giant cell granulomatous reaction with some associated fibrosis (Figures 2 and 3). The granulomatous response was limited to the periphery of the Gore-Tex and although it closely followed the profile of the material it did not encroach into or significantly break up the material. However, some fibrosis penetrated a short way into the Gore-Tex in a few areas. The giant cells were present mainly in a multinucleated form. Figure 4 shows the Gore-Tex strip under crossed Polaroids showing birefringence of the implanted material. Birefringent fragments were only very occasionally seen within isolated giant cells. The giant cell response was mainly seen in areas where the end of a Gore-Tex strip formed a sharp corner. In one area a very small focus of metaplastic bone formation was seen. There was no significant lymphocytic, neutrophilic inflammation or vascular ingrowth.

## Discussion

Gore-Tex has been used as an effective implant for medialization laryngoplasty in the management of paralytic dysphonia and to a lesser extent aspiration [6,7]. Animal studies involving rabbits and a porcine model have demonstrated host tolerance of the implant, with no evidence of granuloma formation, resorption, extrusion or migration of the implant [2,8]. However, adverse responses to Gore-Tex in humans requiring implant removal from the lips, face, nose and larynx have been reported [9-12]. To date, there have been no reports describing the histological changes in a human laryngectomy specimen with a Gore-Tex implant.

## Conclusions

Our findings are consistent with the animal models confirming that Gore-Tex implantation does not result in a significant granulomatous reaction in the human larynx over a 13-month period. Moreover, there is no evidence of resorption or infection. Further, the lack of lymphocytes in association with the granulomas indicates that there is no significant immunological hypersensitivity. Histologically, the slight permeation by connective tissue is similar to that seen in Gore-Tex vascular and cardiac implants. The degree of the slight giant cell response appears to be dependent on the profile of the material; a sharp edge incited more of a response than a flat surface.

## Competing interests

The author(s) declare that they have no competing interests.

## Authors' contributions

All authors equally contributed towards the background research and writing of the paper.

## Pre-publication history

The pre-publication history for this paper can be accessed here:
